# Reversion of Ebolavirus Disease from a Single Intramuscular Injection of a Pan-Ebolavirus Immunotherapeutic

**DOI:** 10.3390/pathogens11060655

**Published:** 2022-06-07

**Authors:** Erin Kuang, Robert W. Cross, Maria McCavitt-Malvido, Dafna M. Abelson, Viktoriya Borisevich, Lauren Stuart, Krystle N. Agans, Neil Mlakar, Arumugapradeep Marimuthu, Daniel J. Deer, William S. Shestowsky, Do Kim, Joan B. Geisbert, Larry Zeitlin, Crystal L. Moyer, Chad J. Roy, Thomas W. Geisbert, Zachary A. Bornholdt

**Affiliations:** 1 Mapp Biopharmaceutical, Inc., San Diego, CA 92121, USA; ekuang@tulane.edu (E.K.); maria.mccavitt@mappbio.com (M.M.-M.); dafna.abelson@mappbio.com (D.M.A.); lauren.stuart@mappbio.com (L.S.); neil.mlakar@zabbio.com (N.M.); aru.marimuthu@mappbio.com (A.M.); william.shestowsky@mappbio.com (W.S.S.); do.kim@mappbio.com (D.K.); larry.zeitlin@mappbio.com (L.Z.); crystal.moyer@eitrbio.com (C.L.M.); 2 Galveston National Laboratory, University of Texas Medical Branch, Galveston, TX 77555, USA; rwcross@utmb.edu (R.W.C.); viborise@utmb.edu (V.B.); knagans@utmb.edu (K.N.A.); djdeer@utmb.edu (D.J.D.); jbgeisbe@utmb.edu (J.B.G.); 3 Eitr Biologics, Inc., San Diego, CA 92121, USA; 4 Department of Microbiology and Immunology, Tulane School of Medicine, New Orleans, LA 70112, USA; croy@tulane.edu; 5 Division of Microbiology, Tulane National Primate Research Center, Covington, LA 70447, USA

**Keywords:** pan-ebolavirus, MBP134, immunotherapeutic, Sudan, SUDV, EBOV, anti-viral, intramuscular, injection

## Abstract

Intravenous (IV) administration of antiviral monoclonal antibodies (mAbs) can be challenging, particularly during an ongoing epidemic, due to the considerable resources required for performing infusions. An ebolavirus therapeutic administered via intramuscular (IM) injection would reduce the burdens associated with IV infusion and allow rapid treatment of exposed individuals during an outbreak. Here, we demonstrate how MBP134, a cocktail of two pan-ebolavirus mAbs, reverses the course of *Sudan ebolavirus* disease (Gulu variant) with a single IV or IM dose in non-human primates (NHPs) as late as five days post-exposure. We also investigate the utility of adding half-life extension mutations to the MBP134 mAbs, ultimately creating a half-life extended cocktail designated MBP431. When delivered as a post-exposure prophylactic or therapeutic, a single IM dose of MBP431 offered complete or significant protection in NHPs challenged with *Zaire ebolavirus*. In conjunction with previous studies, these results support the use of MBP431 as a rapidly deployable IM medical countermeasure against every known species of ebolavirus.

## 1. Introduction

Ebolaviruses are members of the family *Filoviridae* known to cause severe hemorrhagic fever in both humans and nonhuman primates (NHPs), with human mortality rates in some outbreaks approaching 90% [1]. There are now six known ebolavirus species: *Zaire ebolavirus* (EBOV), *Sudan ebolavirus* (SUDV), *Bundibugyo ebolavirus* (BDBV), *Taï forest ebolavirus* (TAFV), *Reston ebolavirus* (RESTV) and *Bombali ebolavirus* (BOMV) [1,2]. To date, two monoclonal antibody (mAb) products, REGN-EB3 (Atoltivimab, Maftivimab, and Odesivimab) and mAb114 (Ansuvimab), have been approved by the U.S. Food and Drug Administration (FDA) for the treatment of EBOV infection. Despite their efficacy against EBOV, these mAbs are not active against the five remaining ebolavirus species [3]. In contrast, ADI-15878 and ADI-23774, the two non-competing mAbs that make up the MBP134 cocktail, target highly conserved non-overlapping epitopes on the ebolavirus glycoprotein (GP) and inhibit GP-mediated membrane fusion across every known species of ebolavirus [4,5,6,7]. The observed mechanism of action behind ADI-15878 and ADI-23774, direct inhibition of GP mediated membrane fusion, likely contributed to the protective potency of these mAbs over other ebolavirus immunotherapeutics. Indeed, we previously demonstrated how a single intravenous (IV) 25 mg/kg dose of MBP134 could reverse the course of ebolavirus disease (EVD) in NHPs challenged with EBOV, SUDV (Boniface variant), or BDBV [4]. To date, no other known ebolavirus immunotherapeutic has matched the protective efficacy of MBP134 in NHP ebolavirus challenge models. Nevertheless, IV administration of a therapeutic in an ebolavirus treatment unit (ETU) is time consuming, resource intensive, and requires medical personnel to deliver the drug within a clinical setting.

A single dose pan-ebolavirus post-exposure prophylactic (PEP)/therapeutic delivered intramuscularly (IM) would dramatically increase the efficiency of treatment in settings with limited medical resources as well as reduce the risk of infection for medical professionals in ETUs. The high dosing (≥50 mg/kg) required for protective efficacy of currently approved EBOV therapeutics necessitates IV administration, limiting rapid large-scale deployment [8]. Additionally, by targeting epitopes limited in activity to only EBOV, both REGN-EB3 and mAb114 fail to address public health threats posed by divergent re-emerging ebolavirus species (e.g., SUDV or BDBV). This shortcoming represents a significant liability also seen with SARS-CoV-2 immunotherapeutics that has led to reduced levels of protection over time [9]. Thus, we set out to determine if MBP134, a highly potent pan-ebolavirus immunotherapeutic, could be successfully administered at lower IV or IM doses. We also examined the impact of serum half-life extensions on the MBP134 mAbs with the hope of increasing their clinical utility for use in prophylaxis or PEP scenarios.

## 2. Results

### 2.1. A single IV Dose of MBP134 Protects NHPs from Lethal SUDV Challenge

**Figure 1:**
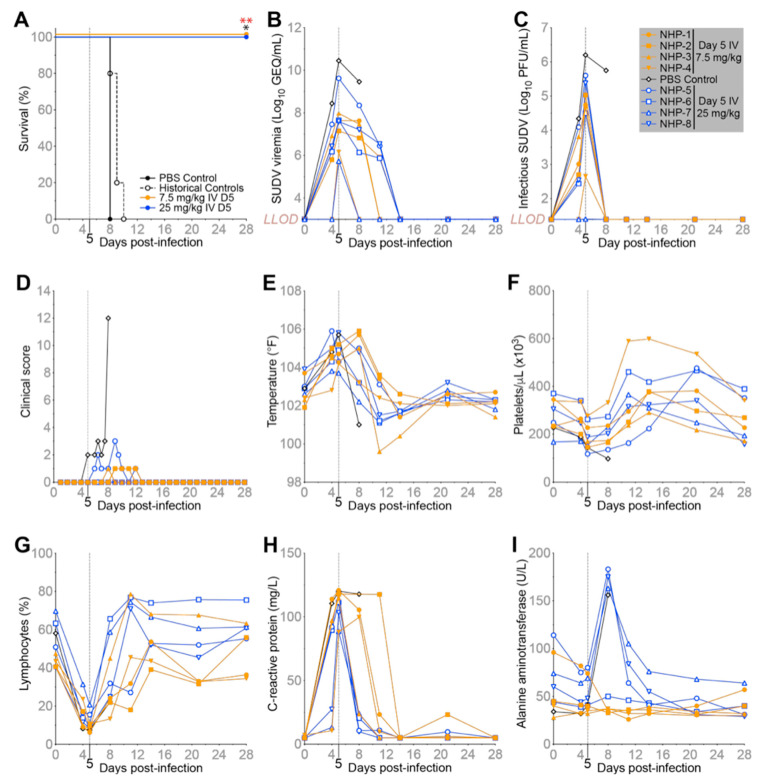
Evaluation of MBP134 delivered IV in NHPs challenged with SUDV. (**A**) Survival curves for NHPs challenged IV with 1000 PFU of SUDV. Animals received either PBS (black), a 25 mg/kg dose (blue), or a 7.5 mg/kg dose (orange) of MBP134 on D5 PI. A log-rank Mantel–Cox test was used to determine *p* values against the in-study control alone (* = 0.0455) or the in-study control combined with historical controls (** = 0.0047, shown in red). (**B**) Average GEQ/mL of SUDV and (**C**) infectious SUDV (PFU/mL) in the blood of each NHP. (**D**) Clinical scores, (**E**) body temperatures, (**F**) platelet counts, (**G**) lymphocyte levels, (**H**) C-reactive protein (CRP) levels, and (**I**) alanine aminotransferase (ALT) levels for each NHP. LOD = limit of detection (3 log10 GEQ/mL and 1.39 PFU/mL). See also Figure S1.

Previously, we demonstrated that a single 7.5 mg/kg dose of MBP134 protected NHPs challenged with SUDV (Boniface variant) [4]. Here, MBP134 was further evaluated for therapeutic efficacy in a fully blinded study in the highly lethal SUDV (Gulu variant) rhesus macaque model. Nine animals were challenged IM with a target dose of 1000 plaque forming units (PFU) of SUDV (actual dose = 1013 PFU). One animal was assigned to the untreated control group with historical control data leveraged to support statistical analyses. The treatment groups (*n* = 4/group) received either a single 25 mg/kg or a single 7.5 mg/kg IV dose of MBP134 on D5 post-infection (PI) (Figure 1A). Both the 25 mg/kg and 7.5 mg/kg dose of MBP134 reversed the course of SUDV disease and protected all of the challenged animals, whereas the control animal succumbed to SUDV infection on D8 PI (Figure 1A). Historical controls receiving the same viral inoculum under identical protocols had a mean time to death of 9 days PI (*n* = 5). Retrospectively, all of the animals were confirmed to be qRT-PCR positive for SUDV infection at time of treatment with viremia ranging from 5.7 to 10.5 log10 genomic equivalents (GEQ)/mL (Figure 1B). Infectious SUDV virus was found in the blood at greater than 4.5 log10 PFU/mL on D5 PI in all but two animals. NHP-4 showed 2.7 Log10 PFU/mL and NHP-7 tested just above the limit of detection (LOD) at 1.4 Log10 PFU/mL on D5 PI (Figure 1C). MBP134-treated animals were PCR negative, or below the LOD, 9 days post-treatment and had no detectable infectious virus or plaque forming units (PFUs) by the first post-treatment collection point on D8 PI (Figure 1B,C). All of the NHPs showed clinical signs of SUDV disease (SVD) as reflected by the presence of fever, reduced platelet count, lymphopenia, elevated C-reactive protein (CRP) levels, and clinical scoring prior to treatment on D5 PI, supportive of a therapeutic indication (Figure 1D–H). Of note, both the control and the 25 mg/kg treatment group displayed elevated levels of alanine aminotransferase (ALT) by D8 PI, returning to baseline levels by D14 PI for the treated animals. In contrast, ALT levels within the 7.5 mg/kg treatment group remained relatively unchanged from baseline (Figure 1I). However, in both treatment groups, aspartate aminotransferase (AST) and alkaline phosphatase (ALP) remained at baseline levels, demonstrating MBP134 inhibited the progression of liver damage typical of SUDV infection as observed in the control (Figure S1). These data demonstrate the single dose therapeutic potency of IV administered MBP134 for the treatment of SUDV infection.

### 2.2. MBP134 Maintains Therapeutic Efficacy When Delivered as an IM Injection

**Figure 2:**
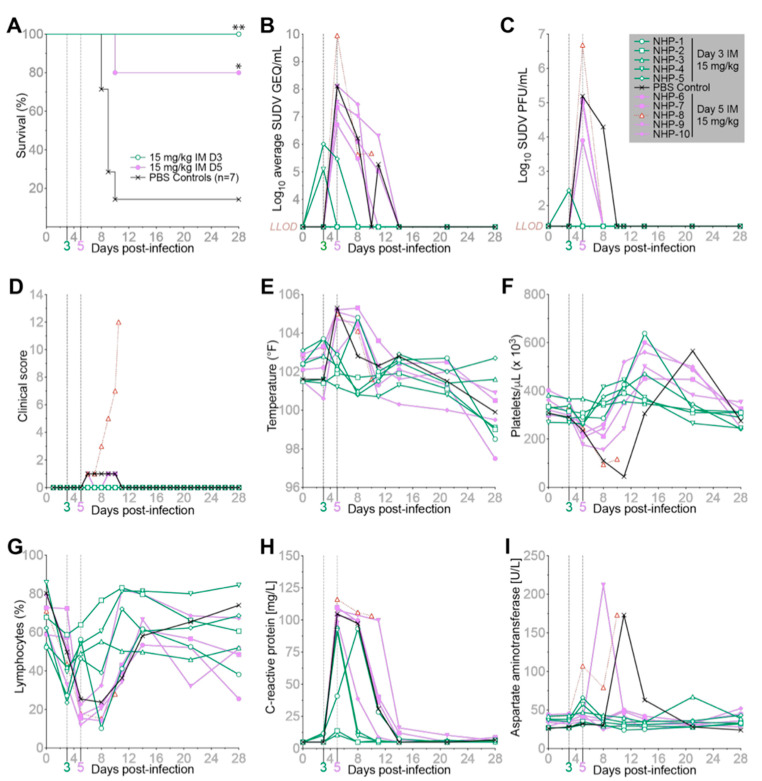
Evaluation of MBP134 as an IM injectable PEP or therapeutic drug in NHPs challenged with SUDV. (**A**) Survival curves for NHPs challenged IM with 1000 PFU of SUDV. Animals received either a 15 mg/kg dose of MBP134 on D3 PI (green) or D5 PI (purple). Historical controls and the in-study control are shown in black. A Log-rank Mantel–Cox test was used to determine *p* values against the combined controls with *p* values of 0.006 (**) and 0.0181 (*) for the D3 and D5 treated animals, respectively. (**B**) Average GEQ/mL of SUDV in the blood of each animal. NHP-8, the only treated animal that succumbed to infection, is shown in red. (**C**) Infectious SUDV (PFU/mL) in the blood of each NHP. (**D**) Clinical scores, (**E**) body temperatures, (**F**) platelet counts, (**G**) lymphocyte levels, (**H**) C-reactive protein (CRP) levels, and (**I**) aspartate aminotransferase (AST) levels for each NHP. LOD = limit of detection (3 log10 GEQ/mL and 1.39 PFU/mL). See also Figure S2.

The complete therapeutic protection observed in NHPs treated with a single 7.5 mg/kg IV dose of MBP134 suggested MBP134 may be sufficiently potent to support administration via IM injection. To evaluate MBP134 as either an IM injectable PEP or therapeutic drug, rhesus macaques were treated with a two-site IM total dose of 15 mg/kg (7.5 mg/kg per site) of MBP134 on either D3 PI (NHP 1–l5) or D5 PI (NHP 6–10). One animal was assigned to the untreated control group, with historical control data leveraged to support statistics (*n* = 1 from the previous study and *n* = 5 from previously published studies [10]). All NHPs were challenged IM with a target dose of 1000 PFU of SUDV (Gulu variant) (actual dose = 888 PFU) using the same viral stock as historical studies [10]. MBP134, when administered as an IM PEP injection on D3 PI, either prevented (NHP 1–3) or reversed (NHP 4–5) the course of SVD, with all of the animals surviving. Similarly, IM therapeutic treatment with MBP134 on D5 PI also provided significant levels of protection (80%; *p*-value = 0.0181) (Figure 2A). Unexpectedly, while the untreated control animal did become viremic and displayed a prolonged course of SUDV disease compared to all of the treated animals (Figure 2), it survived and represents the first untreated NHP (of seven total controls leading up to this study) to have survived a challenge from this SUDV (Gulu variant) viral stock. As anticipated, most of the animals treated D3 PI (NHP 1–3) had no detectable infectious virus and remained RT-PCR negative throughout the study. However, NHP-4 and NHP-5 showed signs of an acute course of SUDV infection with 5.1 and 6.0 log10 SUDV GEQ/mL detected on D3 PI, respectively. Infectious virus was found in NHP-5 on D3 and immediately dropped below the LOD post-treatment. In contrast, all five animals (NHP 6–10) in the D5 treatment group were RT-PCR positive prior to treatment, ranging from 6.7 to 10.0 log10 SUDV GEQ/mL (Figure 2B), and displayed high titers of infectious virus at the time of treatment, ranging from 3.9 to 6.7 Log10 PFU/mL, similar to our first study here and previous SUDV challenge studies [10]. Consistent with the previous experiment, no infectious virus was detectable following MBP134 IM treatment by the next sample collection point on D8 PI (Figure 2C). Prior to treatment, the D5 treatment group registered an onset of fever and thrombocytopenia, whereas most NHPs treated on D3 PI avoided these clinical signs (Figure 2E,F). All of the animals developed lymphopenia and had elevated CRP levels at the time of treatment (Figure 2G,H). Of significance, NHP-8, the only treated animal to succumb to infection, displayed an acute course of SVD compared to the other animals in the study (Figure 2D). Compared to the other animals in the study, NHP-8 had the highest level of viremia at the time of treatment and showed signs of a severe systemic SUDV infection with significant levels of virus (GEQ per gram) present in its lungs, adrenal glands, and kidneys upon necropsy (Figure S2). Additionally, this animal had the highest GEQ/mL and PFU/mL in blood of the cohort prior to treatment on D5 PI (Figure 2B,C). Notably, elevated AST levels appeared much earlier in NHP-8 than in the rest of the cohort, likely impacting the chances of recovery post treatment (Figure 2I). In summary, the data here demonstrate MBP134 can prevent or reverse the course of SVD when administered as a PEP or as a therapeutic via IM injection. 

### 2.3. Half-Life Extension Mutations Expand the Clinical Utility of MBP134

**Figure 3:**
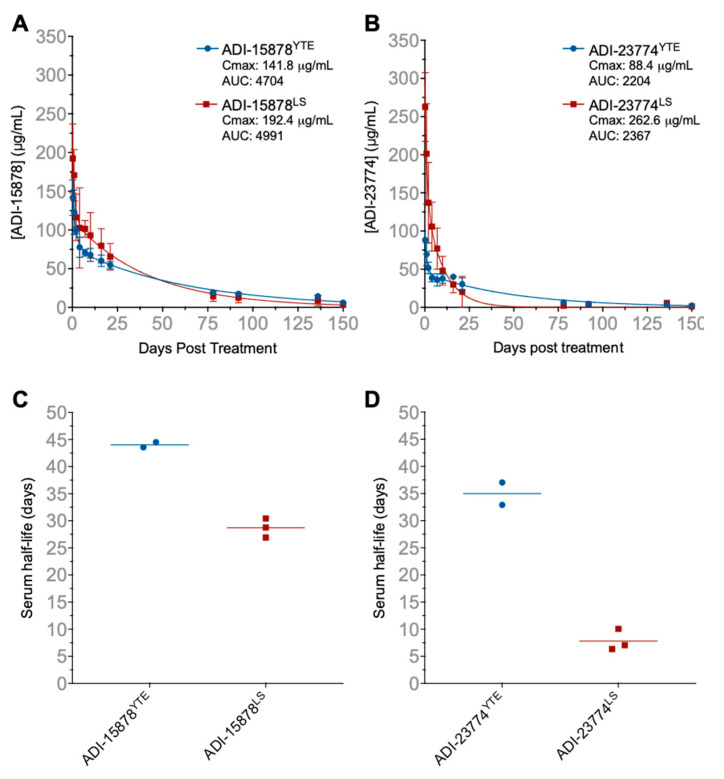
Pharmacokinetic analyses of half-life extension variants of MBP134 in NHPs. (**A**) Serum concentrations of ADI-15878^YTE^ (blue) and ADI-15878^LS^ (red) and (**B**) serum concentrations of ADI-23774^YTE^ (blue) and ADI-23774^LS^ (red) at each collection time point. The maximal concentration (Cmax) and area under the curve (AUC) are displayed in the legends for panels (**A**,**B**). Errors bars represent the mean ± standard deviation. Panels (**C**,**D**) display the calculated serum half-life for each mAb variant in each individual animal.

Previous studies have shown that YTE mutations (M252Y/S254T/T256E) and LS mutations (M428L/N434S) incorporated into an IgG Fc region can significantly extend antibody serum half-life by promoting pH-dependent recycling via binding to human FcRn [11,12]. Studies also suggest these mutations can contribute to increased efficacy by maintaining higher levels of circulating immunotherapies for longer periods of time [13]. Here, the YTE or LS mutations were introduced into the Fc region of ADI-15878 and ADI-23774, creating two half-life extended cocktail variants designated MBP431 and MBP432, respectively. A pharmacokinetics study was conducted in rhesus macaques to determine whether the YTE or the LS mutations were optimal for prolonging MBP134 serum bioavailability. NHPs were given a 14 mg/kg IV dose of either MBP431 (*n* = 2) or MBP432 (*n* = 3). The serum concentration of each antibody was quantified using an anti-idiotype mAb specific for either ADI-15878 or ADI-23774 (Figure 3A,B). The calculated serum half-life of ADI-15878^YTE^ was 44 days, and that of ADI-15878^LS^ was 29 days (Figure 3C). The serum concentration of ADI-15878^LS^ began to drop off at a faster rate than ADI-15878^YTE^ around 50 days post-administration. The half-life of ADI-23774^YTE^ was 34.64 days, and that of ADI-23774^LS^ was 7.1 days (Figure 3D). The serum concentration of ADI-23774^LS^ dropped off dramatically after about 25 days post-administration at a comparatively faster rate than ADI-23774^YTE^. Of note, human clinical studies with human mAbs containing YTE mutations have reported half-lives of 80–112 days [14]. Thus, MBP431 was selected over MBP432 for further evaluation in NHP challenge studies due to its potential to provide months of protection.

### 2.4. MBP431 Provides NHPs Significant PEP and Therapeutic Protection from EBOV

**Figure 4:**
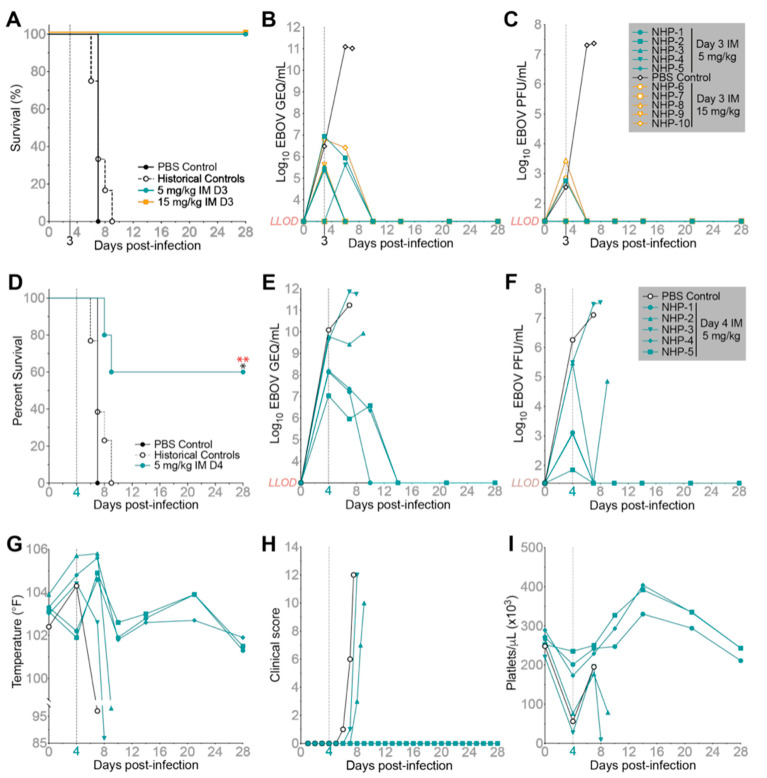
Evaluation of MBP431 as a PEP or therapeutic IM injectable treatment in NHPs challenged with EBOV. (**A**) Survival curves for NHPs challenged IM with 1000 PFU of EBOV. Animals received either a single 5 mg/kg (cyan) or 15 mg/kg (orange) IM dose of MBP431 on D3 PI. Historical controls and the in-study control are shown in black. Statistical significance was determined using a log-rank Mantel–Cox test against the in-study PBS control alone, yielding a *p* value of 0.0253 (*), or combined with 13 historical controls, which yielded a *p* value of 0.0003 for both treatment groups. (**B**) Average GEQ/mL of EBOV and (**C**) infectious EBOV (PFU/mL) in the blood of each NHP treated D3 PI. (**D**) Survival curves for NHPs challenged IM with 1000 PFU of EBOV with the 5 mg/kg IM dose of MBP431 walked out to D4 PI. Statistical significance was determined using a log-rank Mantel–Cox test against the in-study PBS control alone, yielding a *p* value of 0.0253 (*), or combined with 14 historical controls, which yielded a *p* value of 0.0045 (**, red). (**E**) Average GEQ/mL of EBOV and (**F**) infectious EBOV (PFU/mL) in the blood of each NHP treated D4 PI. (**G**) Body temperatures, (**H**) clinical scores, and (**I**) platelet counts for each NHP treated D4 PI. LOD = limit of detection (3 log10 GEQ/mL and 1.39 PFU/mL).

Building on the efficacy demonstrated by MBP134 when delivered IM to NHPs challenged with SUDV, we evaluated the efficacy of MBP431 in NHPs challenged with EBOV. Eleven NHPs were challenged IM with a target dose of 1000 PFU of EBOV (actual dose = 1088 PFU). To evaluate MBP431 as a PEP, the treatment groups (*n* = 5/group) received a two-site IM injection of MBP431 at a total dose of either 15 mg/kg or 5 mg/kg on D3 PI. One control animal received PBS. Both dosing regimens provided complete protection in contrast to the PBS treated control (*p*-value = 0.0003), which met euthanasia criteria on D7 PI (Figure 4A). Historic controls inoculated with the same viral lot under identical protocols had a mean time to death of 7 days PI (*n* = 12). Animals in both groups showed signs of active EBOV infection on D3 PI prior to treatment with virus detected via qRT-PCR from the LOD to 6.8 log10 GEQ/mL. Coinciding with the RT-PCR data, animals displayed levels of circulating infectious virus ranging from the LOD to 3.4 log10 PFU/mL (Figure 4B,C). However, either dose delivered on D3 PI inhibited significant advancement of EBOV infection, with viremia levels remaining below the LOD throughout the remainder of the study. Based on these results, the time to treat was delayed by 24 h to D4 PI in a follow-up study in which 6 NHPs were challenged with EBOV (target dose 1000 PFU, actual dose 1225 PFU). One control animal received PBS, and the other five received a two-site IM injection of MBP431 at a total dose of 5 mg/kg on D4 PI. MBP431 provided significant, but reduced levels of protection, with 60% of treated NHPs surviving (Figure 4D). All of the animals in the study were qRT-PCR positive prior to treatment on D4 PI. Of significance, survivors (NHP-1, NHP-4, and NHP-5) were grouped around 7–8 log10 GEQ/mL, whereas non-survivors (NHP-2, NHP-3, and the control) were grouped at ~10 log10 GEQ/mL on the day of treatment (Figure 4E). Similarly, infectious virus was present in blood samples from all of the animals prior to treatment on D4 PI, with survivors having viral loads up to 3.1 log10 PFU/mL and non-survivors having greater than 5.5 log10 PFU/mL (Figure 4F). Through the course of the study, all of the animals registered an elevated temperature between D4 and D8 PI, with treated survivors returning to baseline temperature by D10 PI (Figure 4G). Clinical scores and severe thrombocytopenia only appeared in non-survivors (Figure 4H,I). Thus, MBP431 can prevent onset of EVD when administered as a PEP and can resolve milder courses of EVD when delivered as a 5 mg/kg IM dose. 

## 3. Discussion

Both the unprecedented 2013–2016 West African ebolavirus epidemic and the ongoing SARS-CoV-2 global pandemic have underscored the need for readily available medical interventions to halt the spread of infectious diseases. During the ebolavirus outbreak in West Africa, as well as more recent outbreaks, the high infection rate experienced by medical staff severely compromised the capability of ETUs to treat patients [15]. This issue was further exacerbated by the time and personnel required for IV infusions in low resource settings. Indeed, 12 patients enrolled in the PALM clinical trial succumbed to EBOV infection prior to receiving any available treatments [8]. A medical countermeasure (MCM) targeting all ebolaviruses that could be rapidly administered via IM injection to instantly prevent or reverse the course of infection could alter the response and treatment paradigms during an ebolavirus outbreak. We evaluated MBP134, a potent pan-ebolavirus therapeutic comprised of two broadly neutralizing human mAbs, ADI-15878 and ADI-23774, as an easily administered IM injectable MCM to treat and/or prevent ebolavirus infection. To expand the clinical utility beyond therapy for potential use as a long-term prophylactic or PEP, we introduced serum half-life mutations into the Fc regions of both mAbs in the MBP134 cocktail. The utility of prophylactics is evident from the success of the prophylactic ring vaccination strategy used during the 2013–2016 EBOV epidemic in Guinea. This strategy was effective due to its ability to stop new infections for 30 days, which protected vulnerable individuals beyond the 10-14 day window during which their immune systems responded to the vaccine [16,17]. In contrast to vaccines, which require varying periods of time to generate protective immunity, a single-dose IM immunoprophylactic could offer instantaneous protection, immediately disrupting the chain of transmission during an outbreak. Of the two half-life mutation variants, YTE and LS, analyzed in our NHP pharmacokinetic studies, we observed that the YTE mutations present in the MBP431 cocktail conferred a longer half-life. Although sequence differences in a mAb variable region can lead to variable serum half-lives in the presence of either the YTE or LS mutations, we cannot rule out the possibility that the particularly short half-life of ADI-23774^LS^ (~7 days) may be due to the development of anti-drug antibodies that were not generated by an equivalent exposure to ADI-23774^YTE^. Ultimately, MBP431 was tested for PEP and therapy and demonstrated significant protective efficacy with a single 5 mg/kg IM dose. With this dosing regimen, MBP431 did not appear to impact NHPs displaying a more advanced state of disease at the time of treatment. Although higher doses of MBP134 administered via IV have already demonstrated complete protection [4], the 5 mg/kg IM dosing of MBP431 tested here represents a potentially realistic single dose IM regimen for an average weight human. This dose is significantly lower than the currently FDA approved mAb treatments, which are 50 mg/kg and 150 mg/kg for mAb114 and REGN-EB3, respectively [18]. It remains to be determined if lower doses of MBP431 tested here in combination with small molecule-based treatments like Remdesivir could amplify protective efficacy in more severe cases via augmented biodistribution, as was observed in recent combination studies for the treatment of SUDV and Marburgvirus infection [19,20]. Future experiments will also focus on the prophylactic utility of MBP431 when administered IM in NHP models of ebolavirus infection.

## 4. Materials and Methods

### 4.1. Study Design

The objective of the research performed here was to determine if a two antibody pan-ebolavirus cocktail administered IM could provide protective efficacy in non-human primates (NHPs) challenged with either SUDV or EBOV, potentially altering ebolavirus outbreak response paradigms in the clinic. For the first study, nine healthy, adult rhesus macaques (*Macaca mulatta*) of Chinese origin (PreLabs) ranging in age from ~4–5 years and weighing 5.4–6.3 kg were challenged IM in the left quadricep with a 1000 PFU target dose of SUDV (Gulu variant). Treatment was initiated 5 days after infection as a single IV dose of MBP134. The duration of this study was 28 days. For the second study, eleven healthy, adult rhesus macaques of Chinese origin (Prelabs) ranging in age from ~4–6 years and weighing 4.0–6.6 kg were challenged IM in the left quadricep with a 1000 PFU target dose of SUDV (Gulu variant). Treatment was initiated 3 or 5 days after infection as a single IM dose of MBP134. In order to maintain blinding of the groups, every animal received an IM injection of either MBP134 or phosphate buffered saline (PBS) on day 3 (D3) and day 5 (D5) from deidentified vials that were randomly assigned prior to challenge. The control received two injections of PBS. The duration of this study was 28 days. For the third study, eleven healthy, adult rhesus macaques of Chinese origin (Prelabs) ranging in age from ~3–4 years and weighing 4.2–5.2 kg were challenged IM in the left quadricep with a 1000 PFU target dose of EBOV (Kikwit variant). Treatment was initiated 3 days after infection as a single IM dose of MBP431. The duration of this study was 28 days. For the fourth study, six healthy, adult rhesus macaques of Chinese origin (Prelabs) ranging in age from ~3–4 years and weighing 4.0–5.2 kg were challenged IM in the left quadricep with a 1000 PFU target dose of EBOV (Kikwit variant). Treatment was initiated 4 days after infection as a single IM dose of MBP431. The duration of this study was 28 days. For all four NHP studies, assignment to each treatment group or control was determined prior to challenge by randomization with an effort made to maintain a balanced sex ratio. All studies were blinded to the research staff. The macaques were monitored daily and scored for disease progression with an internal filovirus humane endpoint scoring sheet approved by the UTMB Institutional Animal Care and Use Committee (IACUC). The scoring changes measured from baseline included posture and activity level, attitude and behavior, food intake, respiration, and disease manifestations, such as visible rash, hemorrhage, ecchymosis, or flushed skin. A score of ≥ 9 indicated that an animal met the criteria for euthanasia. 

### 4.2. University of Texas Medical Branch (UTMB) Ethics Statement

Animal studies were performed in biosafety level (BSL)-4 biocontainment at the University of Texas Medical Branch (UTMB) and approved by the UTMB Institutional Biosafety Committee (IBC) and IACUC. Animal research was conducted in compliance with UTMB IACUC, Animal Welfare Act, and other federal statutes and regulations relating to animals. The UTMB animal research facility is fully accredited by the Association for Assessment and Accreditation of Laboratory Animal Care and adhere to principles specified in the eighth edition of the Guide for the Care and Use of Laboratory Animals, National Research Council.

### 4.3. Expression, Purification, and Formulation of Monoclonal Antibodies from an Engineered CHOK1-AF Cell Line

CHOK1-AF cells stably expressing the ADI-23774 and ADI-15878 mAbs were generated as previously described [4]. Briefly, a dual plasmid system containing expression cassettes for the heavy and light chains of the target mAbs were co-transfected by random integration via chemical means into a modified CHOK1 host cell line (CHOK1-AF) that yields afucosylated glycans on expressed mAbs. Stable selection was initiated with the inclusion of MSX as a selection agent 24 h post-transfection. Upon completion of the final expansion, the culture was maintained in fed-batch for 14 days, after which the supernatant was clarified via filtration and subsequently sterile filtered (0.2 mm) into a 20 L bioprocess bag (Thermo Fisher) prior to protein A purification. For the first NHP SUDV challenge study, MBP134 was formulated for IV delivery in 20 mM sodium citrate, 10 mM glycine, 8% sucrose, 0.01% polysorbate 80 (PS80), pH 5.5 (Buffer 1) at 7.3 mg/mL (low dose treatment) or 24.1 mg/mL (high dose treatment). In order to support IM delivery of MBP134 in the second NHP SUDV challenge study, the drug product was formulated in Buffer 1 at 60.7 mg/mL. MBP431 was first formulated in Buffer 1 at 20.4 mg/mL (low dose) and 60.8 mg/mL (high dose) for the first EBOV challenge study and later formulated in 10 mM histidine, 5% sorbitol, 0.02% PS80, pH 6.0 at 22.4 mg/mL for the final EBOV challenge study. 

### 4.4. Generation of Anti-Idiotype Antibodies

The anti-idiotype antibodies targeting either ADI-15878 or ADI-23774 were raised by Bio-Rad using the HuCal PLATINUM synthetic Fab library [21]. Anti-idiotype Fabs were screened for binding against the target Fab, IgG1, and against a negative IgG1 isotype control antibody yielding the final candidate anti-idiotypes for ADI-15878 and ADI-23774, anti-idiotype-878 (AI878) and anti-idiotype-774 (AI774), respectively. The sequences for each anti-idiotype Fab were provided by Bio-Rad. The Fab heavy and light chains of AI774 and AI878 were individually cloned into a pcDNA3 vector (Invitrogen). A 6x-His tag was added to the C-terminus of each Fab heavy chain sequence. Plasmids containing the Fab heavy chains and light chains were chemically co-transfected into an afucosylated ExpiCHO cell line using the ExpiFectamine CHO Transfection Kit (Gibco). After 9 days of transient expression, the supernatant containing either the AI774 or AI878 Fab was clarified via centrifugation and 0.22 µm filtered prior to purification. The Fabs were purified by affinity chromatography with a 5 mL HisTrap FF crude column (Cytiva). Following loading, the column was washed with PBS + 5 mM imidazole, and the Fabs were eluted with PBS + 250 mM imidazole. For AI774, the nickel column eluate was further purified on a 1 mL HiTrap LambdaFabSelect column (Cytiva). The column was washed with PBS and the AI774 Fab was eluted with IgG Elution Buffer (Pierce). Prior to storage at −70 °C, the AI774 Fab was neutralized to pH 7 with 1 M Tris, pH 8.0.

### 4.5. Pharmacokinetics Study in Rhesus Macaques

A PK study in rhesus macaques was used to determine the serum half-life of the four Fc-engineered MBP134 mAbs to assess their potential as an immunoprophylactic countermeasure. Two NHPs received ADI-23774^YTE^ and ADI-15878^YTE^ (MBP431 cocktail), and three NHPs received ADI-23774^XTND^ and ADI-15878^XTND^ (MBP432 cocktail). Each NHP was given an intravenous dose of 14 mg/kg of the assigned set of mAbs. Blood samples were obtained from the NHPs prior to treatment; 5 h post-treatment; and 1, 2, 4, 7, 10, 16, 21, 78, 92, 136, and 150 d post-treatment. The concentration of each mAb in each serum sample was quantified longitudinally by enzyme linked immunosorbent assay (ELISA) using 96-well half-area ELISA microplates (Greiner Bio-One) coated with 2 µg/mL of mAb-specific anti-idiotype Fab (AI87 or AI47) at 4 °C overnight. The next morning, plates were blocked with 100 µL/well of SuperBlock (Thermo Scientific) at room temperature for 30 min. Serum samples were diluted 1:5000 in 0.05% Tween-20 PBS (PBST) with 1% BSA, and then added to the blocked wells at room temperature for 1 h. Similarly, 12-point standard curves were generated for each mAb by adding 1/3-fold serial dilutions (starting at 3 µg/mL for ADI-23774 and 10 µg/mL for ADI-15878) to the blocked wells. Plates were washed 3 times with PBST, and then incubated with a 1:10000 dilution (in PBST with 1% BSA) of goat anti-human Fc HRP-conjugated antibody (Invitrogen) at room temperature for 1 h. Following 3 washes with PBST, plates were developed by addition of 3,3′, 5,5′-tetramethylbenzidine substrate (KPL SureBlue, SeraCare) and stopped by addition of NH_2_SO_4_. Absorbance values were measured at 450 nm using the Perkin Elmer EnVision multimode plate reader. 

### 4.6. Tulane National Primate Research Center (TNPRC) Ethics Statement

The Tulane University Institutional Animal Care and Use Committee approved all procedures used during this study. The Tulane National Primate Research Center (TNPRC) is accredited by the Association for the Assessment and Accreditation of Laboratory Animal Care (AAALAC no. 000594). The U.S. National Institutes of Health (NIH) Office of Laboratory Animal Welfare number for TNPRC is A3071-01.

### 4.7. Pharmacokinetic Data Analysis

Data obtained from the PK study was analyzed with Prism 8 (GraphPad). Based on separate standard curves generated for each mAb, the serum concentration of each mAb for each timepoint was quantified and plotted against the number of days post-treatment. The serum half-life of each mAb was calculated using nonlinear regression, in which a two-phase exponential decay model (with y constrained to plateau at 0) was used to fit the data. The value reported here is the terminal elimination half-life. 

### 4.8. Challenge Viruses

*Zaire ebolavirus* (EBOV) isolate 199510621 (strain Kikwit) originated from a 65-year-old female patient who had died on 5 May 1995. The study challenge material was from the second Vero E6 passage of EBOV isolate 199510621. Briefly, the first passage at UTMB consisted of inoculating CDC 807223 (passage 1 of EBOV isolate 199510621) at an MOI of 0.001 onto Vero E6 cells (ATCC CRL-1586). The cell culture fluids were subsequently harvested at day 10 post-infection and stored at −80 °C as ~1 mL aliquots. Deep sequencing indicated the EBOV was greater than 98% 7U (consecutive stretch of 7 uridines). No detectable mycoplasma or endotoxin levels were measured (˂0.5 endotoxin units (EU)/mL). *Sudan ebolavirus* (SUDV) isolate 200011676 (strain Gulu) originated from a 35-year-old male patient who had died on 16 October 2000. The study challenge material was from the second Vero E6 cell passage of SUDV isolate 200011676. Briefly, the first passage at UTMB consisted of inoculating CDC 808892 (CDC passage 1 of SUDV isolate 200011676) at an MOI of 0.001 onto Vero E6 cells (ATCC CRL-1586). The cell supernatants were subsequently harvested at day 7 post-infection and stored at −80 °C as ~1 mL aliquots. No detectable mycoplasma or endotoxin levels were measured (˂0.5 EU/mL). Genomic sequencing of the resulting seed revealed a 92.33% 7U genotype prevalence at the glycoprotein editing site [22].

### 4.9. Hematology and Serum Biochemistry

Total white blood cell counts, white blood cell differentials, red blood cell counts, platelet counts, hematocrit values, total hemoglobin concentrations, mean cell volumes, mean corpuscular volumes, and mean corpuscular hemoglobin concentrations were analyzed from blood collected in tubes containing EDTA using a Vetscan HM5 laser-based hematologic analyzer (Zoetis). Serum samples were tested for concentrations of albumin, amylase, alanine aminotransferase (ALT), aspartate aminotransferase (AST), alkaline phosphatase (ALP), blood urea nitrogen (BUN), calcium, creatinine (CRE), C-reactive protein (CRP), gamma-glutamyltransferase (GGT), glucose, total protein, and uric acid by using a Piccolo point-of-care analyzer and Biochemistry Panel Plus analyzer discs (Abaxis). 

### 4.10. RNA Isolation from SUDV-and EBOV-Infected Macaques

On procedure days, 100 μL of blood from K2-EDTA collection tubes was collected prior to centrifugation and was added to 600 μL of AVL viral lysis buffer with 6 μL carrier RNA (Qiagen) for RNA extraction. For tissues, approximately 100 mg was stored in 1 mL RNAlater (Qiagen) for at least 4 days for stabilization. RNAlater was completely removed, and tissues were homogenized in 600 μL RLT buffer and 1% betamercaptoethanol (Qiagen) in a 2 mL cryovial using a TissueLyser (Qiagen) and 0.2 mm ceramic beads. The tissues sampled included axillary and inguinal lymph nodes, liver, spleen, kidney, adrenal gland, lung, pancreas, urinary bladder, ovary or testis, and eye. All blood samples were inactivated in AVL viral lysis buffer, and tissue samples were homogenized and inactivated in RLT buffer prior to removal from the BSL-4 laboratory. Subsequently, RNA was isolated from blood using the QIAamp viral RNA kit (Qiagen), and from tissues using the RNeasy minikit (Qiagen) according to the manufacturer’s instructions supplied with each kit. 

### 4.11. Quantification of Viral Load

Primers and probe targeting the VP30 gene of EBOV and the L gene of SUDV were used for real-time quantitative PCR (RT-qPCR) with the following probes: EBOV, 6-carboxyfluorescein (FAM)-5 = CCG TCA ATC AAG GAG CGC CTC 3 = 6-carboxytetramethylrhodamine (TAMRA); SUDV, FAM-5 = CAT CCA ATC AAA GAC ATT GCG A 3 = TAMRA (Life Technologies, Carlsbad, CA, USA). Viral RNA was detected using the CFX96 detection system (Bio-Rad Laboratories, Hercules, CA, USA) in one-step probe RT-qPCR kits (Qiagen) with the following cycle conditions: EBOV, 50 °C for 10 min, 95 °C for 10 s, and 40 cycles of 95 °C for 10 s and 57 °C for 30 s; SUDV, 50 °C for 10 min, 95 °C for 10 s, and 40 cycles of 95 °C for 10 s and 59 °C for 30 s. Threshold cycle (CT) values representing viral genomes were analyzed with CFX Manager software, and the data are shown as genome equivalents (GEq). To create the GEq standard, RNA from viral stocks was extracted, and the number of strain-specific genomes was calculated using Avogadro’s number and the molecular weight of each viral genome. Virus titration was performed by plaque assay using Vero E6 cells (ATCC CRL-1586) from all plasma samples as previously described [4]. Briefly, increasing 10-fold dilutions of the samples were adsorbed to Vero E6 cell monolayers in duplicate wells (200 μL) and overlaid with 0.8% agarose in 1x Eagle’s minimum essentials medium (MEM) with 5% FBS and 1% P/S. After 6 to 12 days incubation at 37 °C/5% CO_2_, neutral red stain was added, and plaques were counted after 48 h incubation. The limit of detection for this assay was 5 PFU/mL.

## Data Availability

Not applicable.

## Supplementary Materials

The following supporting information can be downloaded at: https://www.mdpi.com/article/10.3390/pathogens11060655/s1, Figure S1: Graphical representation of aspartate aminotransferase (AST) and alkaline phosphatase (ALP) level from the therapeutic evaluation of MBP134 in NHPs challenged with SUDV, Figure S2: qRT-PCR of SUDV genomic equivalents per gram (GEQ/g) present in tissues taken from NHP-8 challenged with SUDV upon necropsy.
