# Supplementary material for: Reversion of Ebolavirus Disease from a Single Intramuscular Injection of a Pan-Ebolavirus Immunotherapeutic
Source: Pathogens. 2022 Jun 7;11(6):655. doi: 10.3390/pathogens11060655 (PMC9228268; doi:10.3390/pathogens11060655)
Supplement: Supplementary file 1 [file pathogens-11-00655-s001.zip › pathogens-1746500-supplementary.pdf]

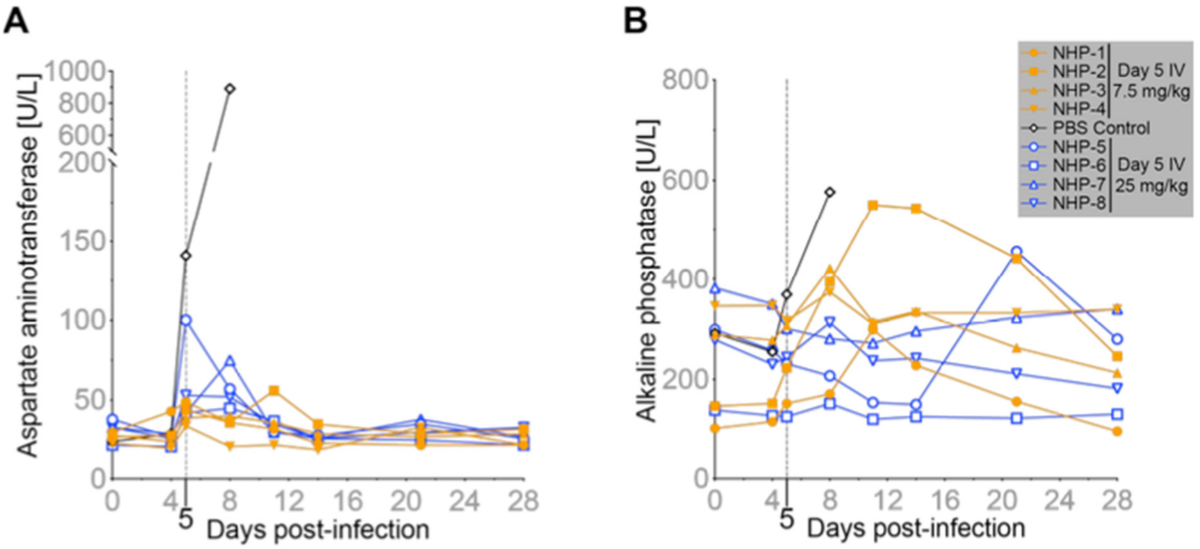

Figure S1. Graphical representation of aspartate aminotransferase (AST) and alkaline phosphatase (ALP) level from the therapeutic evaluation of MBP134 in NHPs challenged with SUDV. AST levels are graphed in panel (A) and ALP levels in panel (B).

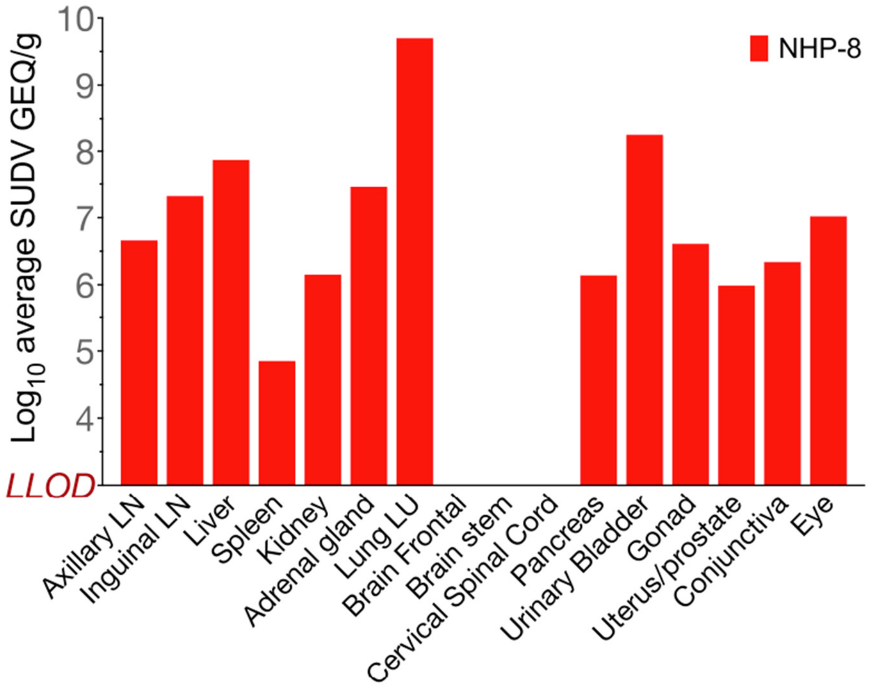

Figure S2. qRT-PCR of SUDV genomic equivalents per gram (GEQ/g) present in tissues taken from NHP-8 challenged with SUDV upon necropsy. The viral load present in emulsified tissue samples at time of necropsy taken from NHP-8 is graphically displayed. NHP-8 had the highest viral load of all Day 5 treated animals and the control animal on D5 PI. That viral load correlates with the severe systemic infection observed in the tissue samples taken from multiple organs in NHP-8.
